# Ataxia in Childhood

**Published:** 1906-05-19

**Authors:** 


### ATAXIA IN CHILDHOOD.

Dr. F. E. Batten, in the issue of "Brain" which covers the autumn and winter quarters of last year, deals with certain rarer varieties of ataxia occurring in the early years of life, that is to say, no consideration is devoted to Friedreich's disease or to the ataxia of cerebellar or mid-brain tumours, or to the ataxia associated with certain cases of diphtheritic paralysis. These rare cases, which are classified as cerebellar diplegias, are divided into three groups. The first group includes cases in which ataxia has been noted early in life, and in which there is a tendency to gradual improvement (congenital cerebellar ataxia). The second group includes cases of ataxia developing suddenly after some acute illness in a child previously quite healthy (acute ataxia, encephalitis cerebelli). The third group consists of children who have been healthy until a certain age, and have then gradually developed ataxia. This variety of disease in some respects resembles Friedreich's disease, but differs from the classical picture of the latter malady (progressive cerebellar ataxia).

The clinical features of congenital cerebellar ataxia are given as follows: "A child whose backwardness in development is evidenced by the late period at which it learns to sit up, to walk, and to talk, is noticed when a few months old to 'tremble' when using its hands: to this, little or no attention is usually paid, but when on sitting up it is noticed that the child shakes its head and the whole trunk is in constant oscillation, some defect is first recognised by the mother and advice is sought. Nystagmus of the eyes on attempting to fix an object may be present. When the child learns to stand, it is noticed that there is great unsteadiness of the legs, and on attempting to walk the legs are thrown about in a most ataxic manner. As the child learns to talk the words are pronounced in a slow, jerky, and monotonous manner. Many of these children are slow eaters, and though they will swallow liquids well, swallow solids very slowly. They are clean in their habits, take an intelligent interest in all their surroundings, and are usually well nourished and have good muscular power. As they grow the ataxia of the arms and legs becomes less, their articulation improves, and in the slightest cases normal development takes place." The type is instanced by the following case. A girl of four years was the first and only child of a mother who had had no miscarriages. Labour had been prolonged, instruments having been used at the birth of the child. She was brought for advice on the score that although four years old she was still unable to walk. She had not learned to use her hands till she was twelve months old, and, though she could talk, her articulation was so defective that it was difficult to understand her. She had always had difficulty in swallowing, and had to be fed very slowly. The difficulty was greater with solids than with liquids. She was intelligent and clean in her habits, and had never had any illness. She was well-formed and well-nourished, able to stand and support her weight on her legs, but unable to walk, and manifested a tendency to fall backwards. When an attempt was made at walking the legs were thrown about in a wildly ataxic manner; the head and trunk were in constant movement, and the hands exhibited a most marked incoördination and intention-tremor. The knee-jerks were brisk and equal. This child gradually improved, until at the age of six years she walked fairly well, the trunk being held in a forward position resembling that adopted in paralysis agitans. The incoördination of the limbs and trunk was still present but much diminished; the knee-jerks remained active, the plantar reflex variable. Intellectual development was good. Dr. Batten considers that the explanation of such symptoms lies in some injury or faulty development of the cerebrum or cerebellum at or before birth. As the cerebrum becomes developed it assumes the coördinating functions of the cerebellum, recovery being more or less complete according to the condition of the cerebral cortex.

The characteristic picture of the second group, acute ataxia, or encephalitis cerebelli, is this. A child who is perfectly healthy and of good intellectual development is attacked by some acute febrile malady, either definite, such as measles, or indefinite. A period of unconsciousness may be present, but general convulsions are rare. After a few days in bed the child appears to proceed to convalescence, but when he is set up in bed he is found to be unable to maintain his balance. There is marked ataxia of the hands and feet, and some affection of the speech. Recovery gradually takes place, but although in a favourable case it may be complete in two or three months, the period of improvement may cover two or three years. Several instances of this type are given in the text; of these the following is the most interesting, because absolute recovery eventually ensued. A boy of four years had had whooping-cough for three weeks, and for this length of time had been observed to exhibit a twitching of the eyes, inability to walk, and a tendency to drop things held in his hands. The chief characteristic of the case was the trembling of the limbs and trunk, which bore a close resemblance to that of disseminated sclerosis. While at rest his body remained free from tremor, but voluntary movement produced a marked ataxia. The fundus oculi was at all times normal. Three months later he was discharged from hospital considerably improved. Two years later he was able to get along on level ground, though in a highly ataxic fashion. "His movements were precisely those of a drunken man." His limbs were strong; he was lively and active, and although he seemed in constant danger of falling he seldom fell. The arms were ataxic, but the tremor of the head and neck was scarcely noticeable. His speech was hesitating, and his voice not properly modulated. Twenty-nine years later he was seen again. He was now a man of 33 years, and a clerk by occupation. No sign of his former malady existed; he was an active man, and included hockey among his recreations.

The features of the third group, progressive cerebellar ataxy, are as follows: A child who has been perfectly healthy and of normal development until a certain age gradually develops an ataxia, which increases in severity until the patient becomes unable to get about. These cases are not, in the author's opinion, instances of Friedreich's disease, although allied to that malady; they are representatives of the group of cerebellar and spinal ataxias. The following is an example. A girl of 11 years had enjoyed good health until her ninth year, when she was observed to be losing the power of walking, while her arms were becoming weak and her speech difficult. On examination she was found to be well nourished and of fair intelligence. The upper central incisors were notched, and the spleen could be felt below the costal margin. There was no further evidence of congenital syphilis. Her articulation was slow and drawling, but all her words were easily understood. There was a marked intention-tremor of both upper and lower extremities. The child could neither stand nor walk without support, and with it was wildly ataxic. The head and trunk shared in the general intention-tremor. There was no optic neuritis, or atrophy, or nystagmus. The pupils reacted to light and accommodation. The knee-jerks were distinctly increased and the arm-jerks brisk. The plantar reflex was variable. The prognosis in the last of these three groups is always bad. The outlook for cases which fall under the head of congenital cerebellar ataxia and of acute ataxia is distinctly hopeful.

